# Virtual reality as a viable alternative to cadaveric dissection for temporal bone surgical training: a pilot crossover trial

**DOI:** 10.1007/s00405-026-10171-6

**Published:** 2026-04-23

**Authors:** Thomas Zheng Jie Teng, David Yong Ming Low, Heng-Wai Yuen, Abhilash Balakrishnan, Celeste Ann Chua

**Affiliations:** 1 Department of Otorhinolaryngology, Head and Neck Surgery, Sengkang General Hospital, Singhealth, Singapore; 2 School of Public Health, Faculty of Medicine, Imperial College London, London, UK; 3 Department of Otorhinolaryngology, Head and Neck Surgery, Singapore General Hospital, Singhealth, Singapore; 4 Department of Otorhinolaryngology, Head and Neck Surgery, Changi General Hospital, Singhealth, Singapore; 5 Ear Nose Throat, Head & Neck Surgery Centre, Mount Elizabeth Medical Centre, Singapore, Singapore

**Keywords:** Virtual reality, Surgical simulation, Temporal bone dissection, Otolaryngology training, Haptic feedback, Cadaveric alternative

## Abstract

**Introduction:**

Surgical simulation has evolved significantly, transitioning from basic models to advanced virtual reality (VR) platforms, addressing the limitations of cadaveric training such as cost, availability, and ethical concerns. VR offers a risk-free, immersive environment for trainees to practice complex procedures, providing real-time feedback and objective performance metrics. Despite its potential, VR applications in Otolaryngology (ENT), particularly temporal bone surgery, remain underexplored due to anatomical complexity and specialty size. The primary hypothesis of this pilot trial was that VR simulation would achieve comparable technical performance to cadaveric dissection, while enhancing self-perceived competency. As a pilot study, no pre-specified non-inferiority margin was established; statistical non-significance was interpreted as potential equivalence requiring validation in a powered trial. This study presents the development and pilot evaluation of a VR-based temporal bone dissection simulator, comparing its efficacy against traditional cadaveric training.

**Methods:**

A single-centre crossover trial involving 14 ENT residents was conducted, with participants randomized into two arms: one starting with VR simulation (VRS) and the other with cadaveric dissection (CD). The VR platform, developed in collaboration with I3 Simulations, incorporated haptic feedback, real-time performance tracking, and scaffolded learning modules. Outcomes were assessed using pre- and post-simulation questionnaires, System Usability Scale (SUS), Technology Acceptance Model (TAM), and Zirkle grading for procedural accuracy.

**Results:**

Results demonstrated a 26.7% increase in self-perceived competency post-VR (*p* < 0.001), with no significant difference in technical performance between VRS and CD (*p* = 0.623). SUS scores indicated moderate usability (score = 65.9 ± 14.2), while TAM scores reflected high acceptability (69.2% ± 12.1%). Qualitative feedback revealed strong trainee endorsement, with 85.7% reporting skill improvement and 78.6% willing to reuse the simulator.

**Conclusions:**

The study supports VR as a viable adjunct or alternative to cadaveric training, offering comparable educational outcomes with added scalability and accessibility. Limitations include small sample size and potential evaluator bias, warranting larger multi-institutional studies. These findings underscore VR’s transformative potential in ENT surgical education, advocating for further refinement and integration into training curricula.

## Introduction

Surgical simulation has undergone a paradigm shift since the 1990 s, transforming from one-layered tissue blocks to multi-layered 3D printed models. Human cadaveric tissue, the gold standard for surgical simulation, has become increasingly difficult to procure due to rising costs and limited availability. Rising demands on current surgical programmes have led to ventures into new approaches to training. Surgical education has increasingly adopted the use of simulation as one of the solutions in the last few years [1]. It offers the opportunity for learners to practise surgical techniques prior to entering the operating theatre, particularly valuable in surgical training because it allows deliberate practice in a safe environment.

Virtual-reality (VR) based surgery simulators are the latest screen-based surgery simulators. VR overcomes ethical, logistical, and financial limitations of cadaveric dissections by providing a risk-free environment where trainees can practise complex procedures. It simulates real-time surgical challenges, including bleeding and tissue movement, while offering tactile feedback and anatomical accuracy [2]. Additionally, VR enables objective performance tracking through metrics like precision and error rates, allowing for personalized training and competency assessment. This adaptability ensures trainees are exposed to the latest techniques and patient-specific data, making VR a scalable and cost-effective solution for modern surgical education.

Despite its potential, VR applications in Otolaryngology (ENT) surgery remain underexplored, creating a significant gap in the literature. Specific to temporal bone drilling, VR simulation would in theory be useful for visualisation of intricate microscopic anatomy and delicate drilling techniques where most junior doctors would not have much hands on experience. However, the development and validation of VR platforms for ENT lag behind other surgical fields, likely due to the complexity of modelling head and neck structures, particularly the temporal bone, and the smaller size of the specialty [3]. The primary hypothesis of this pilot trial was that VR simulation would be non-inferior to cadaveric dissection in improving technical performance, as measured by the Zirkle grading system, while also enhancing self-perceived competency. We present the conception of a VR augmented temporal bone dissection tool and subsequent single centre crossover pilot trial results.

## Materials and methods

### Study setting and participants

This study protocol did not require an institutional review board as determined by institution’s ethics guidelines. The study is reported following the Consolidated Standards of Reporting Trials (CONSORT) guidelines. The pilot study was conducted from August 16 2023 to August 27 2023.

### Software development

The VR software was developed in collaboration with I3 Simulations under the grant InnovPlus Grant Number G.C3.037.001.

The VR surgical training system leverages a multi-stage workflow to enhance anatomical understanding and procedural skills for ENT trainees. The process begins with converting anonymized patient CT scans into detailed 3D models using specialized software (e.g., 3D Slicer, Mimics) [4], which are then optimized for real-time rendering in a virtual environment. These models are integrated into a VR simulation platform, where users interact with them via haptic controllers (Phantom Omni) to experience realistic tactile feedback [5], simulating surgical tool resistance and tissue manipulation. Emphasis was also placed on the ‘”two-handed” technique with virtual suction on the non-dominant hand to simulate actual surgical scenario as closely as possible.

**Fig. 1:**
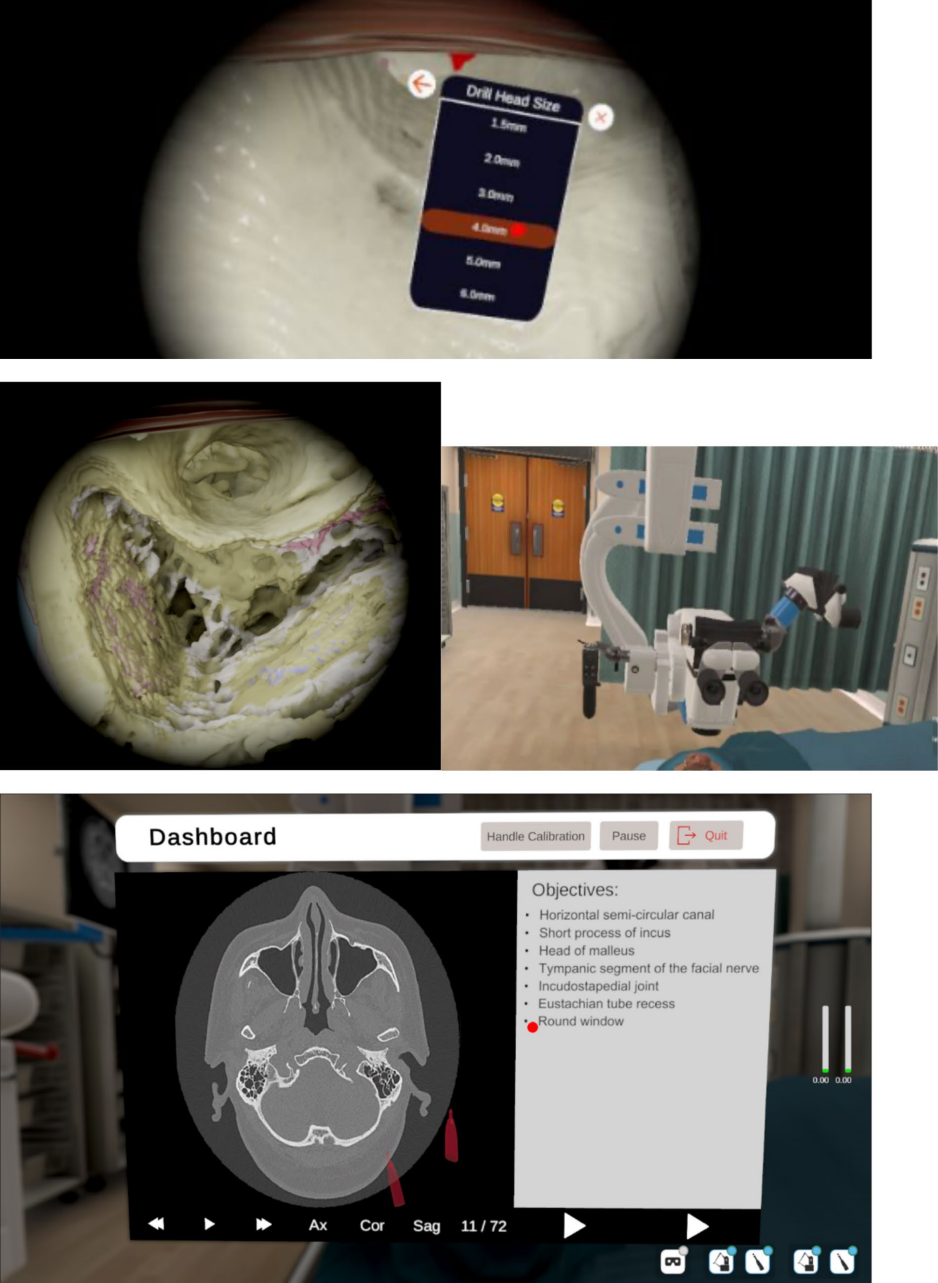
Temporal Bone Simulation Device demonstrating the selection of drill head size (upper photo), simulated temporal bone anatomy (middle left photo), simulated microscope (middle right photo) and simulated dashboard and computer tomography image display for the temporal bone (bottom photo)

The training follows a scaffolded pedagogical design, structured into various modes. Each mode adapts fidelity—psychological (stress/engagement), functional (task reactivity), and physical (visual/haptic realism)—to balance cognitive load and skill acquisition. Performance metrics, including precision, efficiency, and error rates, are captured in real-time, analysed against expert benchmarks, and fed into a Learning Management System (LMS) for personalized feedback [6]. The system’s cloud-based architecture ensures scalability, allowing deployment across institutions while reducing reliance on costly cadaveric training (Fig. 1).

**Fig. 2:**
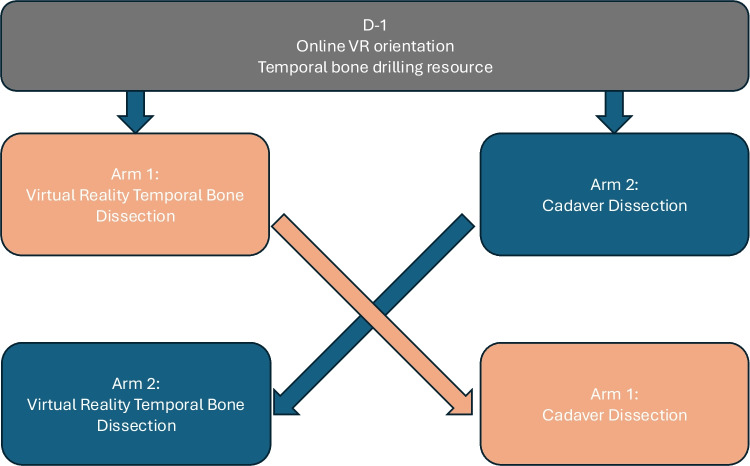
Design trial for the VR Temporal Bone Simulation showing crossover of arms

**Fig. 3:**
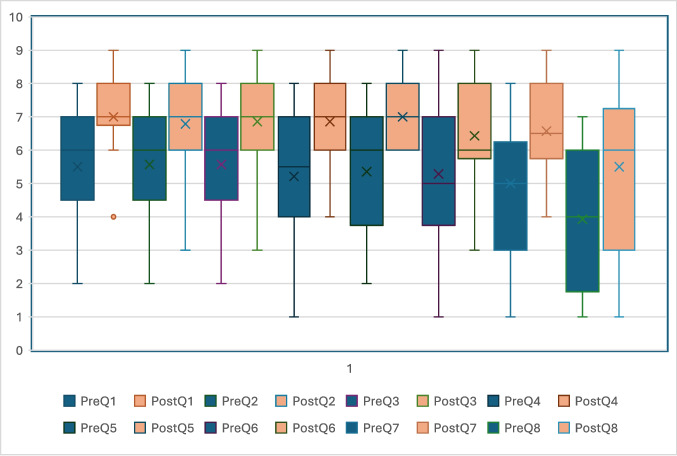
Box and Whisker plot of scores for the pre-VR and post-VR temporal bone questionnaire

During development, subject matter experts assessed the simulator’s fidelity—including physical, functional, and psychological realism—through structured feedback sessions. While formal fidelity metrics were not included in the trial outcomes, expert consensus confirmed that the simulator provided sufficient anatomical and haptic realism to support effective training. The content of the VRS was designed and tested in multiple sessions by subject experts. Three subject experts in the field of Otolaryngology surgery tested the application. Subject experts were presented with a Simulation Station and were given basic orientation of how the software would work, and how to use the provided equipment. Test sessions were also recorded for further review and evidence (Figs. 2 and 3).

The software provided underwent functional testing to minimise the chances of technical issues and bugs affecting the session based around several key topics such as project objective, project requirements, software accuracy and feel of haptic device with the simulation.

Where possible, tests were given a pass/fail with measurable criteria. Subjective feedback (where it is not possible to perfectly digitally replicate reality) had to be agreed by multiple stakeholders and be given clear measurable actions to deliver an outcome.

A separate application was developed purely to allow real-time testing and iteration on the haptic feedback. Subject experts and a member of the development team, and the software allowed the developer to tweak the haptic parameters, allowing rapid iterations on a topic that is fairly hard to explain and partially subjective.

### Development and validation of test instruments

Several instruments were used to measure outcomes. Pre- and post-activity questionnaires to measure self-perceived competency and skills before and after using the VR simulator. This contained 8 questions on a 10-point scale (Appendix A). System Usability Scale (SUS) was adapted and customised for the Otolaryngology setting to measure usability of the simulator [7]. A SUS score of 68 was determined as a benchmark for usability.

Technology Acceptance Model Scale (TAMS) to measure acceptability of the technology to measure the learner’s self-perceived confidence and competence prior to the exercise, post-VR and post-cadaveric dissection [8]. The questions were sub-grouped into questions focusing on perceived usefulness (PU), perceived ease of use (PEU) and behavioural intention to use (BI).

Two Otolaryngology consultants independently graded the cadaveric specimens using the Zirkle checklist. To ensure a strict and comparable assessment to the VR system, the graders were instructed that any structure not clearly identified and annotated by the resident was to be marked as incorrect. Discrepancies between the two consultants were resolved by consensus. Specific tasks and events adapted from the Zirkle grading system were fed into the software to assess performance during the VR simulation to evaluate performance specific to temporal bone drilling [9]. The VR software's automated grading system was programmed using the same Zirkle checklist principles. A structure was only scored as 'correct' if it was positively identified by the user through the system's annotation tool and was within a pre-defined anatomical tolerance. Failure to annotate or incorrect annotation was scored as an error.

### Trial design

Residents from the SingHealth Otolaryngology Residency Programme were recruited voluntarily to evaluate the VR surgical simulator prototype. Residents were divided equally into two groups accounting for their year of residency without blinding. All otolaryngology residents were expected to have baseline theoretical knowledge of temporal bone anatomy and standard otologic surgical approaches appropriate to their training level prior to participating in either training modality. To ensure a standard level of pre-requisite knowledge and familiarity with the VR equipment, each group was required to complete an online introduction module prior to their first dissection session. In the online module, participants were instructed on the required knowledge on temporal bone anatomy, the steps of the three procedures they were to perform (Cortical Mastoidectomy, Facial Recess Approach and Canal Wall Down Mastoidectomy (CWD), key surgical landmarks to be identified as well as the provided information on the setup, calibration and use of the VR simulator. Each group was allocated an arm of the study where they would perform either the VR simulation (VRS) or cadaveric drilling (CD) first (Arm 1 beginning with VRS and Arm 2 beginning with CD respectively). Subsequently, the participant would undergo cross-over and perform the alternate simulation or cadaveric drilling (Fig. 1).

Pre-simulation questionnaires were administered immediately before the participant’s first training modality (either VR or cadaveric dissection). Post-simulation questionnaires were administered immediately after completing the first modality and again after completing the crossover modality.

Performance data from the VR simulator was captured automatically by the software. Cadaveric drilling specimens were graded by physicians using the Zirkle scale (Appendix B). 72 h prior to their first drilling exercise (regardless of VR or cadaveric), participants had to attend a compulsory online module which covered an introduction to the use of the VR goggles and haptic handpieces, a presentation on the steps of the surgical procedures they would have to complete as part of the exercise and an outline on the points they would be assessed and scored on. Qualitative feedback was also obtained from the participants at the end of the exercise.

The participants were instructed to perform three procedures in both the VRS and CD: Cortical Mastoidectomy, Facial Recess Approach and Canal Wall Down Mastoidectomy (CWD).

### Data analysis

Data retrieved was pseudoanonymised and analysed by both SingHealth and i3 Simulations personnel. To account for baseline differences in clinical experience, we performed an exploratory subgroup analysis stratifying residents by training level: junior residents (Year 1–2) versus senior residents (Year 3–5). Given the pilot nature and small sample size (*n* = 14), subgroup comparisons were primarily descriptive and hypothesis-generating, with no inferential statistical claims made. We compared: (1) baseline pre-questionnaire scores, (2) change in self-perceived competency (pre to post), and (3) Zirkle grading performance (both VR-automated and physician-graded cadaveric specimens) between junior and senior groups*.*Two-tailed Students’ T-Test and Fisher’s exact test was used for parametric data as appropriate. Crossover data from both arms were assessed using repeated measures ANOVA crossover, and assessed for normal distribution using Shapiro–Wilk testing. Internal validation of SUS and TAMS scales were performed via Cronbach’s alpha test. Data was analysed via RStudio 2024.12.0 + 467. Significance was placed at p-value of 0.05. Structures that were too damaged to identify on the cadaveric dissection were omitted from analysis when compared to the VR simulation model.

## Results

As a pilot feasibility study, sample size was determined by complete residency cohort inclusion (*n* = 14). Of the 14 residents, 35.7% of the participants were male and had a fairly even distribution of seniority (Table 1). Age for both arms were 31.7 and 32.7 respectively (total range 29–35).

**Table 1:** Table of demographics of residents involved in the study

Demographics			
Variable	Arm 1 (VR First)	Arm 2 (Dissection First)	Total
Age			
Age (Mean (Range))	31.7 (29–34)	32.7 (30–35)	32.2 (29–35)
Gender			
Female (N (%))	6 (42.9%)	3 (21.4%)	9 (64.3%)
Male (N (%))	1 (7.14%)	4 (28.6%)	5 (35.7%)
Residency Year			
Year 1	2	2	4
Year 2	1	2	3
Year 3	2	1	3
Year 4	1	1	2
Year 5	1	1	2

Residents completed pre- and post-simulation questionnaires to rate their self-perceived skill and knowledge competency on a scale of 1–10. The average total pre-score was 41.82 ± 14.82 and the post-score average was 53.0 ± 10.64, representing a 26.7% increase (*P* = 0.00071) (Table 2).

**Table 2:** Table of compute results of the Pre-VR and Post-VR Temporal Bone Questionnaire

Temporal Bone Questionnaire			
Variable	Pre-VR Questionnaire	Post-VR Questionnaire	*p*-value
Overall Scoring (SD)			
Mean	41.82 ± 14.82	53 ± 10.64	0.00071
Mean Questions Scoring (SD)			
Q1: Confidence to perform a full mastoidectomy	5.50 ± 1.74	7.00 ± 1.18	0.0015
Q2: Understanding of surgical anatomy	5.57 ± 1.99	6.79 ± 1.48	0.0108
Q3: Familiarity with surgical equipment	5.57 ± 1.99	6.86 ± 1.41	0.0135
Q4: Confidence in drilling technique	5.21 ± 2.08	6.86 ± 1.41	0.0005
Q5: Confidence identifying critical structures	5.36 ± 1.78	7.00 ± 0.961	0.0013
Q6: Confidence preserving critical structures	5.29 ± 2.13	6.43 ± 1.70	0.0294
Q7: Ability to manage complications (e.g., bleeding)	5.00 ± 2.04	6.57 ± 1.40	0.0014
Q8: Knowledge of procedural steps	3.93 ± 2.27	5.50 ± 2.41	0.0010

### Scoring systems

From the SUS score, Arm 1 scored 62.1 ± 15.2 and Arm 2 scored 69.6 ± 13.2, collectively scoring 65.9 ± 14.2 (p = 0.344) (Table 3). Internal validity was assessed by Cronbach Alpha yielded a value of 0.89 (95% CI 0.78–0.96).

**Table 3:** System Usability Scale (SUS) score of both Arm 1 (VR first) and Arm 2(Dissection first)

Score (mean, SD)	Arm 1 (VR First)	Arm 2 (Dissection First)	Total	*p*-value
System Usability Scale (SUS)	62.1 ± 15.2	69.6 ± 13.2	65.9 ± 14.2	0.344

Cronbach Alpha: 0.89 (0.78–0.96)

TAMS scored yielded varying results for both arms with no significant differences (Table 4). Collectively, the PU yielded 82.2% ± 12.9%, PEU yielded 69.5% ± 17.4% and BI yielded 81.4% ± 16.4%. Overall, the TAMS scored 69.2% ± 12.1%. Discrepancies between structures annotated by participants as assessed by the VR trainer and physicians ranged from 57.1% to 100.0% depending on the structures (Table 5).

**Table 4:** Technology Acceptance Model Scale (TAMS) of both Arm 1 (VR first) and Arm 2(Dissection first)

Score (mean, SD)	Arm 1 (VR First)	Arm 2 (Dissection First)	Total	*p*-value
Perceived Usefulness (PU) (%)	80.2 ± 16.0	84.1 ± 9.6	82.16 ± 12.9	0.586
Perceived Ease of Use (PEU) (%)	63.9 ± 19.0	75.0 ± 15.0	69.46 ± 17.4	0.248
Behavioural Intention to Use (BI) (%)	76.2 ± 86.5	86.5 ± 17.8	81.37 ± 16.4	0.255

Cronbach Alpha: 0.96 (0.92–0.98)

**Table 5:** Discrepancies in annotations scored by VR system and by doctors

	Correctly annotated (VR)	Correctly annotated (Doctor)	Discrepancies*	Accuracy (%)
Zygoma	13	13	1/14	92.9
Henle Spine	11	14	3/14	78.6
Linea	14	13	1/14	92.3
Lateral Semicircular Canal (LSCC)	12	11	5/14	64.3
Tegmen	14	14	0/14	100.0
Sigmoid Sinus (SS)	14	14	0/14	100.0
Sinodural Angle (SDA)	10	9	6/14	57.1
Incus	13	14	1/14	92.9
Buttress**	5	8	3/8	62.5
Round Window (RW)	11	11	3/14	78.6
Facial Nerve (CN7)	14	14	0/14	100.0
Chorda Tympani (CT)**	13	12	1/13	92.9
Incudostapedial joint (ISJ)	9	9	1/9	88.9

^*^Discrepancies defined as incorrectly identified structures, or structures that were not annotated

^**^Cadevers with missing or damaged structures could not be compared with VR simulation and were hence omitted during analysis of data

85.7% of residents reported the VR training improved their skills and 78.6% would use the simulator again and recommend it to others. The self-reported increases were in confidence to start and perform a full mastoidectomy with minimal consultation (24.0–29.0% increase), understanding anatomy (22.4% increase), familiarity with surgical equipment (18.3%), confidence in drilling technique (26.7%), confidence in identifying and preserve critical structures (25.1–26.4%).

### Zirkle grading

Zirkle grading of temporal bone drilling measured by VR and by grading from physicians yielded similar results (Table 6), for both Arm 1 and Arm 2 (VR grading collectively 16.9 ± 2.25 vs grading by physicians 16.4 ± 2.62, p = 0.623). Two-way repeated ANOVA was performed between cadaveric dissection Zirkle scores between Arm 1 (who underwent VR first) and Arm 2 (who underwent CD first). Between each arm of the study, there was no significant difference as well (p = 0.066). The dataset was was normally distributed at each time point (*p* > 0.05) as confirmed by Shapiro–Wilk test. Q-Q plots affirm that the points fall approximately along the reference line.

**Table 6:** Zirkle Grading score for participants based on grading by VR and by doctors

Score (mean, SD)	VR Grading	Doctor Grading	*p*-value
Zirkle Grading			
Arm 1 (VR First)	17.4 ± 2.51	14.7 ± 2.29	0.0793
Arm 2 (Cadaver First)	16.3 ± 1.98	18.0 ± 1.83	0.135
Total	16.9 ± 2.25	16.4 ± 2.62	0.623

### Subgroup analysis by residency seniority

Junior residents (Year 1–2, *n* = 7) had lower baseline pre-questionnaire scores compared to senior residents (Year 3–5, *n* = 7): 31.0 ± 11.9 versus 51.9 ± 8.9 (descriptive comparison). Post-training, both groups demonstrated meaningful improvements in self-perceived competency: junior residents showed a 52.5% increase (31.0 to 47.3) while senior residents showed a 13.2% increase (51.9 to 58.7).

For Zirkle grading performance, senior residents achieved numerically higher mean scores on both VR-automated assessment and physician-graded cadaveric dissection; however, the difference in learning response between modalities (VR versus cadaver) remained non-significant within each subgroup, consistent with the overall cohort findings.​

These descriptive patterns suggest that while senior residents demonstrated higher absolute technical proficiency—likely reflecting greater accumulated clinical exposure—both junior and senior trainees derived comparable educational benefit from VR simulation relative to cadaveric training. The proportionally larger perceived competency gain among junior residents may reflect their steeper learning curve at earlier training stages. Given limited statistical power (*n* = 7 per subgroup), these observations should be considered hypothesis-generating and warrant validation in larger, adequately powered trials.

## Discussion

The integration of VR into surgical training represents a pivotal advancement in medical education, particularly in the context of temporal bone dissection for ENT surgeons [3]. An important principle underscored by this study is that VR simulation, like cadaveric dissection, should be positioned as a complement to—not a substitute for—theoretical instruction. Optimal learning occurs when trainees first acquire foundational anatomical knowledge through didactic teaching, then consolidate this knowledge through hands-on simulation, and finally apply their skills in supervised clinical practice.The data from this study underscores the potential of VR to serve as a viable complement to traditional methods, while also highlighting areas for refinement to enhance its usability and acceptance.

As the demand for innovative, accessible and sustainable training methods grows—VR emerges as a transformative tool that can complement or even substitute traditional cadaveric dissection. The benefits of VR are multifold, including its ability to provide a risk-free, repeatable and highly immersive learning environment. Specific to the Otolaryngology setting, a systematic review by Hudise et al. on the use of VR involving 6 studies showed improvement in hand–eye coordination and spatial awareness in the Otolaryngological surgery setting and did not show any inferiority compared to traditional methods of surgical training [3]. VR not only democratizes access to high-quality surgical training but also aligns with the broader trend of digitalization in medicine, offering a scalable solution to the limitations of cadaveric resources, such as availability, cost, and ethical considerations [10].

Compared to didactic learning of surgical anatomy and procedures, VR learning provides a closer alternative to real life surgery. In the traditional learning model of anatomy, which could be summarized as “2D → 3D → 2D”, learners must imagine 3D structures with 2D cross-sectional images. This process is long and difficult and often results in the memorization of inaccurate or even incorrect 3D structures because of the lack of an ability to immediately correct these representations. While cadaveric dissections offer a hands-on, three-dimensional understanding of temporal bone anatomy, they come with limitations such as scarcity of specimens, high costs, and the inability to simulate pathological variations or surgical complications. As a result, surgical residents who rely solely on 2D images or infrequent cadaveric training often face difficulties in clinical practice, whereas VR bridges the gap between theoretical knowledge and real-world surgical application.

In a randomised control trial by Chen et al. involving 71 surgical residents using 3-dimensional models (3D Models) versus computed tomography to identify anatomical landmarks of abdominal vessels, it was noted that the group who used 3D Models has statistically significantly better scores [11]. This new learning model combines 3D and 2D images and might accelerate the learning process by improving the accuracy of 3D structures and deepening the memory of anatomy. Extrapolating this concept of 3D Models to VR learning, similarly positive results were noted. In a randomised control trial by Lamb et al. involving 38 medical students simulating intramedullary nail surgery, the group that learned via VR had significantly improved time to completion (9.6 min vs 12.2 min, *P* = 0.034) and reduced needs for correction (2.2 vs 2.5; *P* = 0.05) compared to the group who learned via traditional technique guidance, defined as access to study the standard industry surgical technique guide [12]. Given the non-inferiority of VR with promising outlooks on improving visualisation of anatomy, further studies to solidify its benefits in the otolaryngology setting should be pursued.

This study’s findings reveal a statistically significant increase in perceived skill and competency among participants after using the VR simulation, as evidenced by pre- and post-simulation questionnaires. This suggests that subjectively, VR effectively enhances learners’ confidence and self-assessed proficiency, which are critical precursors to technical mastery. Of note, the comparable results on the Zirkle Grading for Temporal Bone Drilling between the VR and cadaver dissection groups demonstrate that VR is non-inferior to traditional methods in achieving technical proficiency. This finding is particularly compelling, as it suggests that VR can replicate the educational outcomes of cadaveric dissection. We note the caveat that in cases where the structure is too damaged to be marker or is absent, the VR simulation may not be able to localise the structure and assumes the candidate to have made an error. Specific to the otology setting, this is corroborated in the recent systematic review by Hudise et al., which affirms the positive potential for VR in otology surgical practice [3]. It is worthwhile noting that the VR technologies applied in the systematic review differ vastly across the studies, from the use of a haptic device drill to digital microscope. The study that parallels our investigation the most would be that of Zhao et al. where a randomised blinded control trial involving twenty trainees in temporal bone dissection using VR or cadaveric dissections revealed significantly better performance scores in the VR group (67% vs 29%; P < 0.001) [13]. In Zhao et al.’s case, the participants were allowed multiple repeated practices on the simulator which could have increased their scores in contrast to our pilot trial allowing participants only a days’ duration of trial. Together, these results affirm the value of VR in modern surgical education while emphasizing the need for ongoing optimization to fully realize its potential.

The lower SUS score in the VR group compared to the cadaver dissection group may reflect the inherent learning curve associated with new technology. Notably, Arm 2, which underwent cadaver dissection first likely benefited from prior anatomical exposure during the cadaveric dissection, thereby finding the VR system more intuitive. This translated to a good SUS score of 69.6, where 68 is usually taken as a benchmark to delineate a 50th percentile or above on the usability of the system [14]. With that being said, there was no statistically significant difference between both arms, albeit possibly from the low power of the study. Possible reasons raised in the qualitative feedback segment of the study that might explain the subpar SUS score include lack of familiarity with the device setup, having insufficient time to familiarise with the product and 3D images causing giddiness after prolonged use.

Conversely, the high scores on the Technology Acceptance Model Scale (TAMS)—particularly in perceived usefulness, ease of use, and behavioural intention to use—highlight the strong potential of VR as a training tool [15]. These high scores likely stem from VR’s ability to provide a controlled, interactive, and visually rich learning environment, which resonates with users seeking innovative and effective educational methods. This echoes the current literature’s views towards the use of VR in surgical training. In a survey by Wang et al. evaluating the use of VR to reduce perioperative anxiety for surgical trainees, it was noted that trainees scored over a factor of 0.7 for the domains in TAMS, similar to our study [16].

As the demand for innovative, accessible, and sustainable training methods grows, VR emerges as a transformative tool that can complement or even substitute traditional cadaveric dissection. The benefits of VR are manifold, including its ability to provide a risk-free, repeatable, and highly immersive learning environment. Specific to the Otolaryngology setting, a systematic review by Hudise et al. on the use of VR involving six studies showed improvement in hand–eye coordination and spatial awareness in the Otolaryngological surgery setting and did not show any inferiority compared to traditional methods of surgical training [3]. VR not only democratizes access to high-quality surgical training but also aligns with the broader trend of digitalization in medicine, offering a scalable solution to the limitations of cadaveric resources, such as availability, cost, and ethical considerations.

Our exploratory subgroup analysis revealed that senior residents (Year 3–5) achieved higher absolute performance scores than junior residents (Year 1–2), consistent with their greater accumulated clinical experience. Importantly, however, the relative educational benefit of VR compared to cadaveric training appeared preserved across both experience levels, with junior residents demonstrating proportionally greater self-perceived competency gains—possibly reflecting steeper learning curves at earlier training stages. Critically, these gains were not limited to subjective confidence but were corroborated by objective Zirkle grading improvements across both modalities, demonstrating that VR simulation enhances residents' actual surgical skills alongside their self-assessed proficiency. This dual benefit—improved technical competency and confidence—positions VR as a valuable tool for developing competent surgeons throughout residency progression, though adequately powered studies are needed to definitively characterize experience-dependent learning trajectories.

Current alternatives in the literature (described in Table 7) specific to temporal bone anatomy dissection remain inadequate due to costs and lack of realism. Cadaveric dissection [17], while considered the gold standard for surgical training, is constrained by limited availability, high costs, ethical concerns, and the inability to allow repeated practice on the same specimen [18]. Non-VR simulators, such as physical models with haptic feedback, offer a more accessible option but often lack the anatomical fidelity and dynamic variability of real bone structures. Specific to temporal bone dissection, the advent of 3-D printing provides customizable and reproducible training tools, yet they may fail to fully replicate the tactile feedback and complex tissue interactions encountered in real surgical scenarios. At present, a small pilot study involving 10 trainees by Frithioff et al [19]. suggests that the use of 3-D printed temporal bone models of varying compositions have been shown to be non-inferior to VR nor cadaveric dissections, albeit a low powered study.

**Table 7:** Pros and Cons table of the current training modality options for temporal bone drilling

Training Modality	Brief Description	Pros	Cons
Cadaveric Dissection [18]	Human cadaver specimens drilled with surgical tools	- Gold standard for realism - Provides authentic haptic feedback - No substitute for actual surgical experience	- Limited availability (ethical, logistical constraints) - High costs - No repeatability on the same specimen
Non-VR Simulators (Physical Haptic Models) [17]	Reusable synthetic bones with force-feedback drills	- Tactile feedback - No ethical issues - Lower cost than cadavers	- Limited anatomic variability - Wear over time - Static anatomy
3D-Printed Temporal Bones [19]	Patient-specific bones printed from CT scans	- Customizable anatomy - Reproducible - No ethical concerns	- Material mismatch (density/texture) - No soft-tissue interaction
VR (Haptic Handpieces) [19]	Virtual drilling with force-feedback stylus	- Realistic drill resistance - Repeatable - Adaptive anatomy	- Costly hardware - Still lacks full cadaveric fidelity

The marriage of augmented reality and physical models has also been described in the literature with promising results. The Stryker Surgical Simulator (S3) – touted as a hybrid temporal bone model with a computer interface that provides tactile and haptic feedback for the user to interact with. A validation study by Meyer et al. shows both residents and consultants providing good feedback on its use [20], albeit with no high powered study comparing it to VR or cadaveric dissection as of yet.

### Cost considerations

While we did not perform formal cost-effectiveness analysis in this pilot trial, the economic implications of VR versus cadaveric training merit discussion. Cadaveric temporal bone training incurs substantial per-specimen costs (procurement, preservation, storage, disposal) plus fixed infrastructure costs (dissection laboratory, microscopes, drilling equipment, ventilation systems) and variable faculty supervision costs. In a study surveying 113 otolaryngology departments in Europe by Frithoff et al., it was noted that cadaveric temporal bones can cost up to $700 per specimen [21]. This cost structure may be particularly advantageous in resource-constrained settings and lesser developed countries, where cadaveric specimen availability is severely limited and laboratory infrastructure is prohibitively expensive. In contrast, VR platforms require higher initial capital investment but substantially lower marginal costs per additional training session, enabling unlimited repeated practice without specimen consumption. However, barriers remain, including upfront hardware costs, technical support requirements, and internet connectivity if needed. Future research should include formal cost-effectiveness analyses comparing total cost per competent trainee across modalities, stratified by setting type (high-resource versus lesser developed countries’ contexts). Hybrid models—combining limited cadaveric sessions for haptic validation with extensive VR practice—may offer optimal cost–benefit ratios as well.

### Limitations

This study is not without limitations, most notably its inadequate statistical power (42% post-hoc) due to the small sample size of 14 residents. This underpowered design increases Type II error risk and prevents definitive conclusions. Ideally, a study of this nature would require at least 36 participants to achieve adequate power, assuming a tolerance for a Type I error (α) of 0.05 and a Type II error (β) of 0.20, which corresponds to 80% power [22]. The small sample size may restrict the generalizability of the findings and their ability to detect subtle differences between the VR and cadaver dissection groups. However, it is important to recognize that this study serves as a pilot test to evaluate the feasibility and preliminary efficacy of VR simulation in temporal bone dissection training. Furthermore, the participant strength was limited as the otolaryngology residency program only had 14 residents whom all participated. This is a recurring issue in such small residency programs as compared to larger residency programs such as General Surgery when investigating interventions. As such, its primary aim was to establish a foundation for future research rather than to provide definitive conclusions.

Another key limitation is the methodological challenge of comparing automated VR-generated Zirkle scores with human-evaluated cadaveric dissection scores. While we implemented a strict grading protocol for the cadaveric dissection to mirror the VR system's logic, inherent differences remain. Therefore, the results should be interpreted as indicative of non-inferior trends rather than direct equivalence. To enhance the reliability and reproducibility of these findings, future studies in larger countries with multiple residency programs could consider expand to include programs’ residents with larger and more diverse cohorts. This would not only strengthen the statistical power but also provide broader insights into the applicability of VR across different educational contexts and learner demographics. Despite these limitations, the study offers valuable preliminary evidence supporting the integration of VR into surgical training curricula.

A further limitation is that we did not prospectively collect standardized time-to-completion data for either the VR or cadaveric dissection sessions. As a result, we could not assess whether one modality conferred advantages in procedural efficiency or learning curve. Future studies should incorporate objective timing metrics, alongside competency and usability outcomes, to better characterise the impact of VR training on operative performance and workflow.

### Future directions

Because the simulator is built on patient CT–derived 3D models, the platform is inherently extensible to anatomical variants and disease-specific temporal bone pathology. In this pilot, we intentionally limited the dataset to normal anatomy to streamline development and evaluation of core functionality. Future iterations will incorporate libraries of variants (e.g., high jugular bulb, low tegmen, aberrant facial nerve course) and pathology (e.g., cholesteatoma, contracted mastoid), enabling surgeons to practice highly individualised, personalised, and challenging patient-specific cases prior to actual surgery. This capability could broaden the simulator's role from generic skills training to targeted preoperative rehearsal and planning, allowing trainees and attending surgeons alike to rehearse complex, real-world scenarios in a risk-free environment.

## Conclusion

In conclusion, this study demonstrates that multi-feedback virtual reality (VR) simulation holds significant promise as a complementary or alternative tool to cadaver dissection for temporal bone surgical training. The data revealed that VR enhances perceived skill and competency, achieves non-inferior technical outcomes compared to cadaveric dissection, and is well-received by users in terms of perceived usefulness and ease of use. While the lower System Usability Score highlights areas for improvement in user interface design, the high Technology Acceptance Model scores underscore the strong potential for VR to be adopted into surgical training programs. Although the study is limited by its small sample size, which restricts its statistical power, it serves as a valuable pilot investigation that lays the groundwork for future research. Expanding this work to include more residents from other institutions to validate these findings and further refine VR technology. As surgical education continues to evolve, VR represents a transformative innovation that can address the limitations of traditional platforms, offering a scalable, accessible, and immersive learning experience for the next generation of ENT surgeons.

## Data Availability

Additional data may reasonably be requested from the corresponding author.
